# The Impact of Virtual Streamer Anthropomorphism on Consumer Purchase Intention: Cognitive Trust as a Mediator

**DOI:** 10.3390/bs14121228

**Published:** 2024-12-20

**Authors:** Chunyu Li, Fei Huang

**Affiliations:** 1 Seoul Business School, aSSIST University, Seoul 03767, Republic of Korea; lichunyu@stud.assist.ac.kr; 2 School of Digital Commerce, Heilongjiang Polytechnic, Harbin 150070, China

**Keywords:** virtual streamer, anthropomorphism, cognitive trust, purchase intention, live streaming e-commerce

## Abstract

As an important tool for brand promotion and marketing, the status of virtual streamers is gradually improving, especially in the Chinese market with a huge Internet user base. Virtual streamer anthropomorphism has gradually become an important research content in the field of consumer behavior. However, the specific mechanism by which the multidimensional anthropomorphic characteristics of virtual streamers affect consumer trust and purchase intention requires further investigation. Therefore, based on the avatar theory, this research explores how the anthropomorphic characteristics of virtual streamers affect consumer purchase intention through cognitive trust. The analysis was performed using SPSS 27.0 and AMOS 24.0, establishing a structural equation model. Through the analysis of questionnaire data from 503 Chinese consumers, it was found that behavioral anthropomorphism, cognitive anthropomorphism, and emotional anthropomorphism all exert a notable influence on cognitive trust. Appearance anthropomorphism and emotional anthropomorphism directly affect purchase intention, and cognitive trust has a significant impact on purchase intention. Moreover, cognitive trust fully mediates the effects of behavioral anthropomorphism and cognitive anthropomorphism on purchase intention and partially mediates the effects of emotional anthropomorphism on purchase intention. This study enriches the application of avatar theory in virtual streamers in live e-commerce and provides theoretical backing for virtual streamer development and enterprise marketing strategies. It also offers practical insights to help brands optimize virtual streamers and improve consumer participation and purchase conversion rates.

## 1. Introduction

As digital technology has advanced rapidly in recent years, virtual streamers have become widely utilized in e-commerce live streaming, establishing themselves as a vital tool for brand marketing. Virtual streamers can establish interactive relationships with consumers, bridging the gap between brands and consumers and thus demonstrating significant market potential [1,2]. In China, by 2024, the Internet user population is estimated to approach 1.1 billion, with the penetration rate reaching 78.0%. The number of online video users has grown to 1.068 billion, while e-commerce users have reached 905 million. Additionally, the number of live streaming users has approached 800 million [3]. This highlights China’s vast Internet user base and the promising prospects of its live streaming e-commerce market. The metaverse and virtual reality, as two prominent innovations, are transforming the digital landscape by merging the real and virtual worlds to create immersive experiences for audiences. Within the metaverse, the value of e-commerce is projected to reach an estimated USD 2.6 trillion [4].

As artificial intelligence and animation technologies have progressed, digital characters have been widely applied in scenarios such as social media and e-commerce live streaming. Research shows that the anthropomorphic features of virtual characters can enhance users’ willingness to interact and emotional resonance, thereby promoting engagement between users and digital personas [5,6]. Within live stream e-commerce settings, virtual streamers, as a new form of interaction, are gradually becoming key factors in enhancing consumer engagement and increasing purchase intention [7]. Anthropomorphic design can enhance users’ sense of trust and willingness to participate by simulating social interactions, which is particularly significant in the process of interaction between virtual streamers and consumers [8]. Social media influencers, in contrast to traditional celebrity endorsements, offer greater credibility due to their closer connection with users, providing a reference for researching virtual streamers [9].

Although existing research has increasingly concentrated on applying streamer attributes within live stream e-commerce settings, the majority has examined the features of human streamers operating on live or social media platforms. There is still limited exploration of how the multidimensional features of virtual streamers influence audience trust and buying intention [10]. Furthermore, studies have indicated that characteristics such as the likability and reactivity of virtual streamers can significantly enhance buying intention by increasing users’ sense of presence. This highlights the significant role of virtual streamers in e-commerce live streaming, specifically in promoting purchasing behavior by enhancing user interaction experiences [11].

Therefore, this research seeks to explore how the four anthropomorphic dimensions of virtual streamers, namely appearance anthropomorphism, behavioral anthropomorphism, cognitive anthropomorphism, and emotional anthropomorphism, affect consumer purchase intention through cognitive trust, thereby filling this research gap.

This research uses a questionnaire survey to gather data from Chinese consumers and utilizes structural equation modeling (SEM) to examine the associations among different anthropomorphic dimensions, cognitive trust, and purchase intention, along with testing mediating effects. The study provides three key contributions. First, we extend avatar theory into the realm of virtual streamers, detailing the distinct impact pathways of the four anthropomorphic dimensions of appearance, behavior, cognition, and emotion on consumer trust and purchase intention. Secondly, by introducing cognitive trust as a mediating variable, we reveal how the various anthropomorphic characteristics of virtual streamers indirectly or directly influence consumers’ purchasing decisions, providing practical guidance for brands in design and communication approaches for virtual streamers. Finally, this research establishes a theoretical foundation for future exploration of the application of virtual streamers in different cultural contexts and product types, expanding the application scenarios of anthropomorphism and trust theory in digital marketing.

The structure of this study is organized as follows: Section 2 presents a thorough discussion of the theoretical foundation and research hypotheses, including an introduction to avatar theory, a discussion on the anthropomorphism of virtual streamers, and a literature review on cognitive trust and purchase intention. It concludes with the formulation of research hypotheses and the development of the research model. Section 3 elaborates on the research methodology, encompassing the study design, measurement of variables, and data collection methods. Section 4 presents the empirical analysis, including reliability and validity tests, common method bias examination, path analysis, and mediation effect analysis. Section 5 focuses on discussion and conclusions. This section identifies the limitations of the study and proposes directions for future research. It also provides a detailed discussion of the results, highlighting their theoretical and practical implications. Finally, the section concludes with a comprehensive summary of the entire study.

## 2. Theoretical Background and Research Hypotheses

### 2.1. Avatar Theory and Virtual Streamers’ Anthropomorphism

Virtual figures have become increasingly vital in contemporary marketing strategies, particularly as digital technology advances, as companies are gradually adopting virtual avatars for brand endorsement to innovate marketing approaches. Avatar theory is widely applied in advertising and brand promotion, positing that virtual avatars not only influence users’ emotional experiences and social interactions but also shape consumers’ perceptions of brands [12,13].

Research has found that virtual self-discrepancy (the gap between users’ self-image in virtual environments and their real self) significantly diminishes users’ self-presence and immersion. Additionally, virtual other-discrepancy (differences in the appearance of virtual others) indirectly impacts users’ trust and interaction smoothness by influencing their social presence [14]. Virtual avatars also demonstrate potential for enhancing user immersion and encouraging positive behaviors. For instance, virtual characters in games can motivate users to adopt healthier lifestyles in real life [15], and personalized avatars have been shown to help users alleviate anxiety [16].

The effectiveness of avatars depends on their realism in form and behavior, encompassing basic avatars, visually realistic but functionally limited avatars, intelligent avatars, and fully human-like digital avatars in both appearance and intelligence. Different avatar designs affect purchase intentions in various ways, offering companies guidance for applications across diverse contexts [17]. In metaverse environments, highly realistic avatars strengthen users’ sense of self-presence and emotional connection, thereby increasing their intention to use [5]. In summary, virtual avatars significantly influence consumer behavior, brand perception, and emotional management, providing fresh strategic insights for marketing in domains such as real-time e-commerce.

In the development of the Brand Anthropomorphism Scale (BASC), brand anthropomorphism was categorized into four dimensions: appearance, moral virtues, cognitive perception, and emotional awareness. Validation studies indicated that these dimensions positively influence brand trust and commitment [18]. Additionally, anthropomorphism involves assigning human-like traits, motivations, or emotions to non-human agents, making AI assistants more personable and prompting positive social and psychological responses from users [19]. A virtual avatar, meanwhile, is a digital entity with an anthropomorphic appearance, controlled by humans or software, and capable of interaction. Its design elements include appearance realism and behavioral realism [17]. Similarly, virtual humans are highly human-like characters, including virtual influencers, streamers, and idols. Despite their rapid development, how users perceive these anthropomorphized characters remains unclear [20]. Furthermore, virtual influencers’ anthropomorphic characteristics are divided into four key aspects: appearance, moral virtues, cognitive awareness, and emotional sensitivity, representing the human-like attributes of virtual entities [6]. Building on prior studies, this research classifies virtual streamer anthropomorphism into four categories: appearance anthropomorphism, behavioral anthropomorphism, cognitive anthropomorphism, and emotional anthropomorphism.

### 2.2. Virtual Streamers’ Anthropomorphism and Cognitive Trust

Cognitive trust represents a type of trust based on rational evaluation, relying on objective judgments of another’s competence, reliability, and professionalism, with a focus on the ability to fulfill responsibilities and behavioral consistency [21]. In e-commerce contexts, cognitive trust stems from consumers’ assessments of the platform’s expertise, goodwill, and integrity, helping them mitigate risks in an environment of information asymmetry [22]. Without direct interpersonal interaction, users establish cognitive trust through rational assessments of merchants’ reliability and honesty, which is crucial in their purchasing decisions [23]. Consumers typically form cognitive trust in service providers by evaluating their reputation and track record, thus enhancing the stability of service relationships [24]. Within real-time e-commerce environments, cognitive trust depends on viewers’ assessments of a streamer’s expertise and reliability; for instance, a streamer’s timely response strategies can significantly strengthen this trust [25]. The essence of cognitive trust lies in evaluating others’ professional quality and task performance, which provides a stable trust foundation, especially in highly uncertain collaborative contexts [26]. Additionally, in live streaming, consumers’ cognitive trust in a CEO derives from perceived expertise and reliability, fostering brand acceptance and engagement [27]. Research suggests that quality of interaction, empathy, and anthropomorphic psychological traits are key factors in enhancing AI device acceptance in the service industry. When AI devices demonstrate empathy and high-quality interactions, particularly by understanding and responding to user needs, trust and acceptance levels increase significantly, facilitating everyday use [28]. Anthropomorphic features such as a human-like voice, cognitive intelligence, and interaction etiquette have also been shown to enhance social presence, thereby increasing trust, interaction frequency, user reliance, and engagement [29]. Studies indicate that for low-cost environmental behaviors, highly anthropomorphic virtual influencers who appear similar to the audience increase trust and engagement intention; conversely, for high-cost behaviors, appearance dissimilarity is more effective in enhancing trust and engagement [30]. Accordingly, this study proposes the following hypotheses:

**H1:**  *Virtual streamer anthropomorphism positively affects cognitive trust.*

**H1a:**  *Appearance anthropomorphism positively affects cognitive trust.*

**H1b:**  *Behavioral anthropomorphism positively affects cognitive trust.*

**H1c:**  *Cognitive anthropomorphism positively affects cognitive trust.*

**H1d:**  *Emotional anthropomorphism positively affects cognitive trust.*

### 2.3. Virtual Streamers’ Anthropomorphism and Purchase Intention

Virtual streamers’ characteristics impact consumers’ purchase intention by enhancing social and transmission presence. Specifically, virtual streamers’ likability and responsiveness directly increase purchase intention and also indirectly enhance it by improving social and transmission presence. Moreover, the vitality of virtual streamers further contributes to purchase intention indirectly [11]. Additionally, virtual influencers’ anthropomorphic features positively impact purchase intention by increasing trustworthiness and parasocial bonds, particularly as ethical values and cognitive awareness significantly boost consumer trust and purchase intentions [6]. Accordingly, the study proposes the following hypotheses:

**H2:**  *Virtual streamer anthropomorphism positively affects purchase intention.*

**H2a:**  *Appearance anthropomorphism positively affects purchase intention.*

**H2b:**  *Behavioral anthropomorphism positively affects purchase intention.*

**H2c:**  *Cognitive anthropomorphism positively affects purchase intention.*

**H2d:**  *Emotional anthropomorphism positively affects purchase intention.*

### 2.4. Cognitive Trust and Purchase Intention

In e-commerce, cognitive trust is considered a core driver of purchase intention. Without face-to-face interaction, users establish trust through a platform’s professionalism, security, and merchant integrity, which increases their willingness to make purchases [23]. Cognitive trust is mainly influenced by utilitarian value, such as saving time, reducing costs, and improving efficiency, which effectively promotes purchase intention [31]. In metaverse shopping scenarios, higher cognitive trust allows consumers to make purchasing decisions with greater confidence, particularly impacting older consumers, while younger consumers are more driven by emotional trust [32]. Drawing from these insights, this hypothesis is formulated:

**H3:**  *Cognitive trust positively affects purchase intention.*

### 2.5. Mediating Role of Cognitive Trust

Consumers’ intrinsic and extrinsic motivations for online shopping can influence their purchase intentions through cognitive trust [31]. In addition, studies have shown that the physical attractiveness and attitude homophily of social network influencers impact their credibility, which, in turn, positively influences users’ intentions to purchase niche products [10]. Within live stream e-commerce, cognitive trust serves as a mediator between a streamer’s response strategies and audience feedback. Positive response strategies enhance cognitive trust, stimulating positive word-of-mouth, while avoidance strategies decrease trust, leading to negative feedback [25]. Cognitive trust is also a crucial mediator between streamer characteristics and impulsive buying behavior, increasing the credibility of the streamer’s information, reducing decision uncertainty, and prompting consumers to make quicker purchase decisions [33]. In virtual streamer broadcasts, anthropomorphic features make virtual streamers easier to understand and trust. Cognitive trust converts consumers’ initial impressions into trust in the streamer’s recommendations, thereby increasing purchase intention [7]. Building on the above, the study suggests the following hypotheses:

**H4:**  *Cognitive trust mediates the effect of virtual streamer anthropomorphism on purchase intention.*

**H4a:**  *Cognitive trust mediates the effect of appearance anthropomorphism on purchase intention.*

**H4b:**  *Cognitive trust mediates the effect of behavioral anthropomorphism on purchase intention.*

**H4c:**  *Cognitive trust mediates the effect of cognitive anthropomorphism on purchase intention.*

**H4d:**  *Cognitive trust mediates the effect of emotional anthropomorphism on purchase intention.*

### 2.6. Research Model

**Figure 1:**
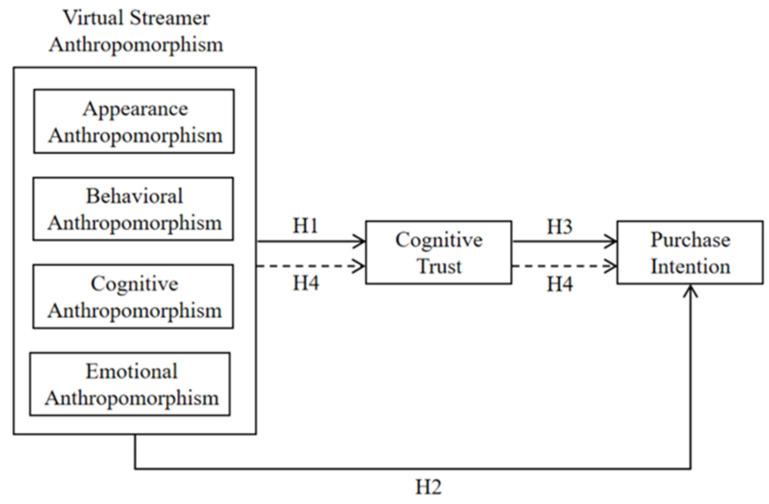
Research model.

Overall, this study examines how virtual streamer anthropomorphism influences purchase intention through cognitive trust. As shown in Figure 1, the research model categorizes virtual streamer anthropomorphism into four dimensions as independent variables: appearance anthropomorphism, behavioral anthropomorphism, cognitive anthropomorphism, and emotional anthropomorphism. Cognitive trust in virtual streamers serves as the mediating variable, and purchase intention as the dependent variable.

## 3. Research Methodology

### 3.1. Research Design

This study used SPSS 27.0 and AMOS 24.0 software to analyze the data. First, SPSS 27.0 was employed to conduct a reliability analysis and exploratory factor analysis (EFA) on the survey data. The reliability analysis, using Cronbach’s Alpha, assessed the internal consistency of the scales, while the EFA examined the relationships between items and latent variables, providing a robust structural foundation for a subsequent confirmatory factor analysis (CFA). Next, AMOS 24.0 was used to perform the CFA, evaluating the model’s fit and calculating the composite reliability (CR) and average variance extracted (AVE) to verify convergent validity. Discriminant validity was confirmed by comparing the square roots of the AVE with the correlations between constructs. To address potential common method bias, a single-factor test was conducted using AMOS. The results indicated that the single-factor model’s fit was significantly worse than that of the measurement model, suggesting that common method bias did not pose a significant concern. Furthermore, SPSS was employed to perform multicollinearity diagnostics. The variance inflation factor (VIF) values for all variables were below 3, indicating the absence of multicollinearity issues. Finally, using SEM in AMOS, the research analyzed the path relationships between the anthropomorphic characteristics of virtual streamers and consumers’ purchase intentions. Additionally, the mediation effect of cognitive trust was tested through the Bootstrap method to thoroughly validate the research hypotheses. The adoption of SEM was particularly suitable for this study as it allowed for precise analysis of the complex causal relationships among latent variables and quantified the mediating role of cognitive trust in the relationship between virtual streamer anthropomorphism and consumers’ purchase intentions.

This study focuses on virtual streamers and selected Chinese consumers as the research sample for the following reasons. First, by 2024, the live streaming user base in China will surpass 700 million, representing 70.6% of all Internet users. This indicates that China’s live streaming e-commerce market is vast. Moreover, virtual digital humans are widely applied in China’s live streaming e-commerce sector. Many companies have launched virtual live streaming hosts who introduce products and engage in live broadcasts [3]. Secondly, virtual streamers have gained notable recognition within the cultural context of China. Numerous virtual streamers with significant fan bases have emerged on Chinese live streaming and video platforms, suggesting that Chinese audiences are receptive to technological innovation and virtual characters [34]. Based on the rapid growth of China’s live streaming e-commerce industry and the rising popularity of virtual streamers, this study utilizes data collected from Chinese consumers for analysis.

### 3.2. Variable Measurement

The development of the measurement scale in this study followed a systematic process comprising several stages.

First, the initial measurement items were designed by referencing established scales from relevant literature. All items were evaluated using a 5-point Likert scale ranging from 1 (“strongly disagree”) to 5 (“strongly agree”). During the initial questionnaire design phase, feedback was solicited from several scholars in the field of management, and the questionnaire was continuously refined and optimized based on their input. For items adapted from English-language scales, a rigorous process of translation and back-translation was conducted multiple times to enhance linguistic accuracy and conceptual alignment.

Secondly, a pilot study was conducted to screen and optimize the items. Using SPSS 27.0, an exploratory factor analysis was performed on the pilot survey data. Items with low factor loadings or insufficient alignment with the intended constructs were removed, resulting in an optimized formal scale.

Finally, SPSS 27.0 was used to analyze the valid data from the formal survey to verify the reliability and validity of the finalized scale. At this stage, no items were removed from the formal scale. The finalized measurement items are presented in Table 1.

### 3.3. Data Collection Process

This study distributed an electronic questionnaire to Chinese respondents via an online survey platform (http://www.wjx.cn). A pilot survey was conducted first, targeting users who were familiar with or had watched virtual streamers. A total of 79 valid responses were collected. Based on the analysis of the pilot survey data, the researchers refined the questionnaire items to develop the formal questionnaire.

The formal questionnaire began with an introduction and instructions for respondents. To ensure the relevance of the sample, a screening question was included: “Are you familiar with or have you watched live streams hosted by virtual streamers?” Respondents who answered “No” were excluded. Additionally, attention-check questions were embedded in the questionnaire to further ensure data quality. Responses failing these checks were excluded from the analysis.

The formal survey was launched in October 2024 and remained open for approximately one month. After applying the screening criteria, a total of 503 valid responses were collected.

Table 2 presents the descriptive statistics of the sample. Among the 503 respondents, 46.5% were male, and 53.5% were female. The largest age group was 26–35 years old, accounting for 41.7% of the total sample, while respondents aged 18–35 comprised 68.7%, indicating that live streaming e-commerce users are predominantly young. In terms of educational attainment, 58.8% of the respondents held a bachelor’s degree or higher. Regarding monthly income, 46.9% reported an income between RMB 5001 and 10,000.

Overall, the distribution of sample characteristics aligns closely with patterns observed in prior research [36,37,38]. These findings suggest that the sample effectively represents the core user group of China’s live streaming e-commerce market—young individuals with higher education levels and disposable income. This alignment underscores the representativeness of the sample within the target population, providing a reliable data foundation for this study.

## 4. Data Analysis and Results

### 4.1. Reliability and Validity Analysis

SPSS 27.0 was used to evaluate the internal consistency of the scales through reliability analysis. The results indicated that the Cronbach’s Alpha values for all constructs were above 0.7 (refer to Table 3), with appearance anthropomorphism at 0.825, behavioral anthropomorphism at 0.870, cognitive anthropomorphism at 0.866, emotional anthropomorphism at 0.894, cognitive trust at 0.892, and purchase intention at 0.871, indicating good reliability of the scales [39,40].

Subsequently, the data were analyzed for validity using SPSS. The results are shown in Table 3. Bartlett’s test of sphericity showed significant results (χ^2^ = 8520.214, df = 325, *p* < 0.001), and the KMO value was 0.953, indicating that it is suitable for the next factor analysis [41]. According to the EFA of the data using SPSS, the factor loadings of all measurement items exceeded 0.6 (see Table 3). This shows that each measurement item has a significant correlation with the construct to which it belongs and has strong explanatory power for the construct.

Confirmatory factor analysis (CFA) was conducted in AMOS 24.0 to confirm the structural validity of the measurement model. The model fit was good, with specific indicators shown in Table 4: χ^2^ = 389.554, χ^2^/df = 1.372, RMR = 0.032, RMSEA = 0.027, GFI = 0.943, AGFI = 0.930, NFI = 0.955, RFI = 0.949, IFI = 0.987, TLI = 0.986, and CFI = 0.987, all meeting the recommended standards and indicating good model fit.

In addition, this study tested the standardized coefficients in AMOS, and the results showed that the standardized coefficients of all measurement items were significant (*p* < 0.001). The coefficients and significance levels are shown in Table 5. Convergent validity was evaluated through composite reliability (CR) and average variance extracted (AVE). As shown in Table 5, all constructs had CR values exceeding 0.7, and the AVE values were above 0.5, satisfying the criteria for convergent validity [41]. This indicates that the measurement items exhibit good consistency at the construct level and sufficiently explain the latent variables.

To assess discriminant validity, the square root of each construct’s AVE was compared against correlations with other constructs. Table 6 demonstrates that each construct’s AVE square root surpasses its correlations with other constructs, confirming strong discriminant validity for the scales [42].

### 4.2. Common Method Bias Test and Multicollinearity Analysis

As the data were collected through a survey, there was a possibility of common method bias. To address this concern, we conducted a confirmatory factor analysis of a single-factor model using AMOS [43]. Specifically, we constructed a model where all measured items loaded onto a single common factor to assess the fit of this single-factor model. The results indicated that the fit indices of the single-factor model (χ^2^/df = 8.312, RMR = 0.086, RMSEA = 0.121, GFI = 0.669, AGFI = 0.612, NFI = 0.714, RFI = 0.689, IFI = 0.739, TLI = 0.716, and CFI = 0.738) were worse than those of the measurement model, suggesting that common method bias was not a serious issue in this study. To further assess the presence of multicollinearity among the independent variables in the model, we analyzed the data using SPSS and calculated the variance inflation factor (VIF) [44]. The results showed that the VIF values for all independent variables ranged from 1.804 to 2.186, all of which were less than 3, indicating that there was no serious multicollinearity problem in this study.

### 4.3. Path Analysis and Hypothesis Testing

SEM in AMOS 24.0 was used to analyze the relationships among virtual streamer anthropomorphism, cognitive trust, and purchase intention, and to test the hypotheses [45]. Table 7 presents the results.

This study hypothesized that the appearance, behavioral, cognitive, and emotional anthropomorphism of virtual streamers would positively influence consumers’ cognitive trust (H1a, H1b, H1c, and H1d). The analysis results indicate that behavioral anthropomorphism (β = 0.314, *p* < 0.001), cognitive anthropomorphism (β = 0.181, *p* < 0.01), and emotional anthropomorphism (β = 0.306, *p* < 0.001) significantly affect cognitive trust, supporting H1b, H1c, and H1d. However, appearance anthropomorphism does not significantly affect cognitive trust (β = 0.062, *p* = 0.258), thus not supporting H1a. Additionally, the anthropomorphic characteristics of virtual streamers were hypothesized to positively influence purchase intention (H2a, H2b, H2c, and H2d). The results show that appearance anthropomorphism (β = 0.241, *p* < 0.001) and emotional anthropomorphism (β = 0.243, *p* < 0.001) have a significant influence on purchase intention, supporting H2a and H2d. Conversely, the influence of behavioral anthropomorphism (β = 0.062, *p* = 0.375) and cognitive anthropomorphism (β = 0.089, *p* = 0.145) on purchase intention is not significant, thus not supporting H2b and H2c.

Finally, the positive effect of cognitive trust on purchase intention (H3) is supported (β = 0.243, *p* < 0.001).

### 4.4. Mediation Effect Test

This research employed SEM in AMOS 24.0 and used the Bootstrapping method to test cognitive trust as a mediator between appearance, behavioral, cognitive, and emotional anthropomorphism and purchase intention [46]. Detailed analysis results are shown in Table 8.

The indirect effect of appearance anthropomorphism on cognitive trust is not significant (Est. = 0.015, *p* = 0.264, BC 95% CI = [−0.013, 0.054]), indicating that appearance anthropomorphism does not enhance purchase intention through cognitive trust, and therefore, H4a is not supported. The indirect effect of behavioral anthropomorphism is significant (Est. = 0.076, *p* < 0.001, BC 95% CI = [0.031, 0.149]), while its direct effect on purchase intention is not significant (*p* = 0.416), indicating that cognitive trust fully mediates the link between behavioral anthropomorphism and purchase intention, supporting H4b. The indirect effect of cognitive anthropomorphism is significant (Est. = 0.044, *p* = 0.004, BC 95% CI = [0.014, 0.094]), and its direct effect is not significant (*p* = 0.191), indicating that cognitive trust also fully mediates the relationship between cognitive anthropomorphism and purchase intention, supporting H4c. The indirect effect of emotional anthropomorphism is significant (Est. = 0.074, *p* = 0.001, BC 95% CI = [0.032, 0.132]), and there is a significant direct effect on purchase intention (Est. = 0.243, *p* = 0.002), indicating that cognitive trust partially mediates the link between emotional anthropomorphism and purchase intention, supporting H4d.

In summary, behavioral and cognitive anthropomorphism have a full mediating effect on purchase intention through cognitive trust, while emotional anthropomorphism has a partial mediating effect, and appearance anthropomorphism has no significant mediating effect [47]. This suggests that appearance anthropomorphism alone is insufficient to enhance cognitive trust; rather, behavioral and cognitive characteristics are crucial, with emotional anthropomorphism also moderately increasing purchase intention.

## 5. Discussion and Conclusions

### 5.1. Limitations and Future Research Directions

This research has certain limitations that merit exploration in future studies. First, the participants in this study were predominantly individuals familiar with or who had watched virtual influencers. While this sampling approach ensured the relevance of the sample, China is a vast country with diverse regions, economic backgrounds, and consumer habits, which may lead to varying levels of acceptance of virtual streamers. Future research could expand the sample coverage by considering consumers from different regions, age groups, and cultural backgrounds. Furthermore, this study concentrated on examining cognitive trust as a mediating factor and did not delve deeper into the potential impact of other variables such as affective trust. Future studies could look into the influence of other mediating variables or consider the moderating effects of individual characteristics such as gender or age to conduct a more comprehensive study of the trust mechanism between virtual steamers and consumers. Finally, the data for this study were collected through a survey. Future research could consider conducting case studies or experiments to investigate related issues or utilize machine learning to analyze the content of live streams.

### 5.2. Discussion on Results

This study empirically investigated the underlying mechanism through which the anthropomorphic characteristics of virtual streamers influence consumer purchase intention via cognitive trust. Our findings revealed that different dimensions of anthropomorphism have varying impacts on cognitive trust and purchase intention. Notably, cognitive trust plays a significant mediating role in some paths. These results not only validate existing theories but also provide new insights into the field of virtual streamer research.

Firstly, appearance anthropomorphism did not significantly influence cognitive trust. As research has shown, not all virtual character appearances can evoke consumer trust and reliance [48]. This finding suggests that a basic human-like appearance alone is insufficient to directly stimulate consumers’ trust in virtual streamers. Humanoid entities need to influence the social relationships that users can perceive, such as social presence, in order to increase users’ trust in them [8]. In previous studies, the effect of anthropomorphism on trust was not a direct relationship, which is consistent with the conclusion of this paper [7]. This study further found that appearance anthropomorphism can directly have a significant impact on consumers’ purchase intention. This finding reveals that the action path of appearance anthropomorphism may bypass the trust mechanism and directly affect purchase motivation. This may be because the appearance design conforms to consumers’ aesthetics or preferences, so it can directly increase purchase intention, which is consistent with the results of prior research [49].

Secondly, behavioral anthropomorphism has a significant positive impact on cognitive trust. This shows that users are more likely to have a certain degree of trust in virtual streamers with humanized interactive behaviors. Virtual streamers are a form of artificial intelligence, which is an intelligent technology that can interact with the surrounding environment and simulate human behavior [50]. This anthropomorphism of artificial intelligence has an impact on trust. Designers often consider the human-like qualities of robots when designing robots to influence trust [51]. However, the behavioral anthropomorphism of virtual streamers has no direct effect on consumers’ purchasing intention, which may indicate that virtual streamers mainly meet users’ entertainment or social needs, and it is difficult for simple behavioral anthropomorphism to directly stimulate consumers’ purchasing intention [52]. Therefore, this reveals that the human-like behavior of virtual streamers can obviously narrow the distance between them and consumers and make consumers trust them, but this is not a direct factor in promoting purchases.

Thirdly, cognitive anthropomorphism has a significant positive impact on cognitive trust, which shows that when virtual streamers have certain logical characteristics or knowledge characteristics, they can effectively gain consumer trust [53]. This is consistent with previous research results. Artificial intelligence technology is applied in many fields. In the study of intelligent agents, the level of anthropomorphism can indeed significantly improve users’ cognitive trust [54]. However, cognitive anthropomorphism does not show a significant impact on purchase intention. This may be because consumers regard virtual streamers as an information provider or tool. Therefore, the cognitive anthropomorphism of virtual streamers does not constitute a factor in direct inducement of purchasing behavior [55].

Fourthly, emotional anthropomorphism has a significant positive impact on both cognitive trust and purchase intention, which shows that if virtual streamers are given the ability to express emotions, it will not only enhance users’ trust in the streamer but also directly affect purchasing behavior through emotional resonance. In previous studies, researchers studying smart voice assistants found that users’ trust in voice assistants was highly correlated with the intimacy between the user and the device, and that the level of anthropomorphism was also moderately correlated with trust [56]. The anthropomorphism of chatbots can affect perceived trust and emotional evaluation, which in turn affects perceived enthusiasm and purchase intention [49]. Therefore, we extend this mechanism of correlation between artificial intelligence and user emotions to the characteristics of virtual streamers’ emotional anthropomorphism, showing that virtual streamers’ emotional anthropomorphism affects trust and can play an important role in consumer purchasing decisions.

Furthermore, the findings revealed that cognitive trust positively influences purchase intention, consistent with prior studies [31,57]. This underscores the pivotal role of trust in virtual streamer live broadcasts. When verifying the mediating role of cognitive trust, it was found that cognitive trust plays a complete mediating role in the impact of behavioral anthropomorphism and cognitive anthropomorphism on purchase intention, while it plays a partial mediating role in the impact of emotional anthropomorphism. This is consistent with existing research, which agrees that with the rapid growth of the number of Internet users, trust is particularly important among the many factors that affect online purchase intentions. In social media marketing, brand trust can have an impact on consumer purchasing decisions, and trust can also regulate perceived anthropomorphism [38,52,58,59]. However, on the path of appearance anthropomorphism, the mediating effect of cognitive trust is not significant, which shows that although appearance anthropomorphism enhances the attractiveness of virtual streamers, it is difficult for consumers to develop a sense of trust based on the appearance characteristics of virtual streamers alone.

### 5.3. Theoretical Implications

First, this study addresses a research gap concerning virtual streamers’ anthropomorphism’s effect on purchase intention. The existing literature primarily examines how virtual imagery influences user experience, with limited systematic analysis of how anthropomorphic features influence purchasing decisions. This study explores the mechanism by which anthropomorphic features affect purchase intention through cognitive trust, providing a new perspective for academia. Secondly, this research broadens the application and measurement of avatar theory. By categorizing virtual streamers’ anthropomorphism into four dimensions of appearance, behavior, cognition, and emotion and validating their varying effects on purchase intention, this classification offers a more detailed framework for future measurement and reveals the complex mechanisms of anthropomorphism across various contexts. This research also presents cognitive trust as a mediator, emphasizing its key role in how virtual streamer anthropomorphic traits affect purchase intention. The results indicate that cognitive trust fully mediates behavioral and cognitive anthropomorphism and partially mediates emotional anthropomorphism, enhancing trust theory applications. Overall, this study broadens the application boundaries of anthropomorphism and trust theories, providing theoretical support for research on virtual streamers and practical guidance for their design.

### 5.4. Practical Implications

Firstly, this research offers actionable guidance for designing virtual streamers. The results show that the multidimensional anthropomorphic characteristics of virtual streamers have different effects on consumer trust and purchase intention, with emotional, cognitive, and behavioral traits significantly enhancing cognitive trust. Companies should prioritize emotional expression and cognitive–behavioral traits when optimizing virtual streamers to strengthen connections with consumers and improve the credibility and effectiveness of product recommendations. Secondly, this study offers insights for brand marketing strategies. Cognitive trust serves as a mediator between virtual streamer anthropomorphism and purchase intention, suggesting that companies can indirectly boost consumer purchase intention by enhancing the professionalism and reliability of virtual streamers. Therefore, companies should focus on creating a consistent and professional image for virtual streamers to ensure reliable information delivery, thereby strengthening brand trust and loyalty. Additionally, this study provides a new perspective for customer relationship management. Cognitive and behavioral anthropomorphic traits promote purchase intention by enhancing cognitive trust. Companies can leverage the anthropomorphic characteristics of virtual streamers to reinforce customer relationships, for example, by optimizing interactive design to increase emotional intelligence and responsiveness, thereby boosting consumer engagement and trust. Finally, this study offers empirical support for AI-driven virtual marketing technologies. The findings show that virtual streamers’ anthropomorphic traits positively influence consumer behavior, with cognitive trust playing a mediating role. This provides a scientific basis for further innovation in virtual marketing technologies and encourages companies to focus on anthropomorphic traits and building trust to enhance user experience and explore new business models.

### 5.5. Conclusions

With the booming development of the digital economy, live streaming e-commerce has become a new frontier for brand marketing. In this field, virtual streamers, as an emerging interactive tool, can connect with consumers and enhance the shopping experience by simulating human characteristics or behaviors. China, as one of the world’s largest Internet markets, has seen a rapid development of live streaming e-commerce, providing a rich practical scenario for the study of virtual streamers. However, although virtual streamers have shown great potential in attracting consumer attention and promoting purchasing behavior, systematic research on how their anthropomorphic characteristics affect consumer psychology and behavior is still limited.

The existing literature mostly focuses on the technical implementation and initial user reactions of virtual streamers and lacks in-depth discussions on how the anthropomorphic characteristics of virtual streamers specifically affect the process of building consumer trust and forming purchase intentions. Especially under the mediating role of cognitive trust, the mechanism of how the anthropomorphic characteristics of virtual streamers affect consumer purchasing decisions has not yet been clarified. This research gap limits our comprehensive understanding of the role of virtual streamers in e-commerce live broadcasts and also restricts brands from effectively using virtual streamers to formulate marketing strategies. Therefore, based on the avatar theory, this study empirically analyzed the impact of the personification of virtual streamers on cognitive trust and consumer purchase intention. Through structural equation modeling analysis of questionnaire data from 503 participants in China, we found that the behavioral anthropomorphism, cognitive anthropomorphism, and emotional anthropomorphism of virtual streamers significantly improved cognitive trust. Appearance anthropomorphism and emotional anthropomorphism can directly enhance purchase intention, while cognitive trust has a significant impact on purchase intention.

The innovation of this paper is that it is the first to subdivide the anthropomorphic characteristics of virtual streamers into the above four dimensions and link these four dimensions with the mediating role of cognitive trust, providing a new perspective for understanding how consumers build trust with virtual characters and how these characters promote consumer purchase intention. This study also has some limitations, such as the geographical limitations of the sample and the specificity of the research design. Future research can expand the sample range, explore the impact of virtual streamer anthropomorphism in different cultural backgrounds, and consider other possible mediating variables or control variables. In addition, the use of a longitudinal research design or experimental methods may further verify and deepen the findings of this study.

The research results are of great significance for understanding and designing virtual streamers suitable for live e-commerce activities. Theoretically, this research expands the application of avatar theory in the field of digital marketing, particularly concerning how virtual streamers influence consumer trust and purchasing behavior. On a practical level, this study provides empirical support for how brands can design virtual streamers to optimize consumer engagement, promote consumer purchases, and improve the competitiveness of virtual streamers. In terms of specific practical suggestions, companies or brands can consider developing advanced interactive functions for virtual streamers, such as implementing responses to consumers’ personalized questions and interacting with consumers in a vivid and real way during live broadcasts to increase consumers’ immersive experience. In addition, the image, actions, and expressions of virtual streamers can be tailored to align with the preferences of their target audience to enhance appeal. Virtual streamers can also be used to convey brand values and product information, ensure the consistency and transparency of information, gain consumers’ favor, and build consumer trust.

## Figures and Tables

**Table 1:** Measurement items.

Variables	Items	Reference
Appearance Anthropomorphism	A1 The virtual streamer looks human-like.	[7,18]
	A2 The virtual streamer resembles a real human.	
	A3 The virtual streamer has a human-like appearance.	
Behavioral Anthropomorphism	B1 The virtual streamer’s movements appear natural.	[7,28]
	B2 The virtual streamer’s voice sounds natural.	
	B3 The virtual streamer has freedom of action.	
	B4 The virtual streamer has decision-making ability.	
Cognitive Anthropomorphism	C1 The virtual streamer has consciousness.	[19,28]
	C2 The virtual streamer has a mind of its own.	
	C3 The virtual streamer is creative and has imagination.	
	C4 The virtual streamer is capable of reasoning.	
Emotional Anthropomorphism	E1 The virtual streamer has its own emotions.	[7,18]
	E2 The virtual streamer feels remorse for actions it deems shameful.	
	E3 The virtual streamer can empathize with people who feel down.	
	E4 The virtual streamer feels guilt when it hurts someone.	
	E5 The virtual streamer feels shame when people have negative views and judgments about it.	
Cognitive Trust	CT1 The virtual streamer is trustworthy.	[33,34]
	CT2 I believe what the virtual streamer says.	
	CT3 The virtual streamer is reliable.	
	CT4 There is no need to worry at all when dealing with the virtual streamer.	
	CT5 I believe in the expertise and capabilities of the virtual streamer.	
Purchase Intention	PIN1 I would purchase the products promoted by the virtual streamer during the live streaming.	[34,35]
	PIN2 I intend to purchase the products promoted by the virtual streamer during the live streaming.	
	PIN3 I would make the virtual streamer’s live streaming my preferred shopping channel.	
	PIN4 I am willing to recommend the products promoted by the virtual streamer to my friends and family.	
	PIN5 I plan to frequently use the virtual streamer’s live streaming for shopping in the future.	

**Table 2:** Demographic information of the participants (*n* = 503).

Characteristic	Category	Frequency	Percentage (%)
Gender	Male	234	46.5
	Female	269	53.5
Age (years)	18–25	136	27
	26–35	210	41.7
	36–45	128	25.4
	45 or above	29	5.8
Education level	High school or below	57	11.3
	Associate degree	150	29.8
	Bachelor’s degree	224	44.5
	Master’s degree or above	72	14.3
Average monthly income	RMB 3000 or below	39	7.8
	RMB 3001–5000	112	22.3
	RMB 5001–10,000	236	46.9
	Above RMB 10,000	116	23.1

**Table 3:** Reliability and exploratory factor analyses.

Construct	Items	Factor Loading	Cronbach’s Alpha
Appearance Anthropomorphism	A1	0.755	0.825
	A2	0.698	
	A3	0.788	
Behavioral Anthropomorphism	B1	0.708	0.870
	B2	0.752	
	B3	0.677	
	B4	0.654	
Cognitive Anthropomorphism	C1	0.755	0.866
	C2	0.74	
	C3	0.675	
	C4	0.743	
Emotional Anthropomorphism	E1	0.761	0.894
	E2	0.748	
	E3	0.702	
	E4	0.703	
	E5	0.709	
Cognitive Trust	CT1	0.759	0.892
	CT2	0.681	
	CT3	0.702	
	CT4	0.733	
	CT5	0.752	
Purchase Intention	PIN1	0.786	0.871
	PIN2	0.707	
	PIN3	0.713	
	PIN4	0.669	
	PIN5	0.719	
KMO			0.953
Bartlett’s Test	Approx.χ^2^		8520.214
	df		325
	Sig.		0.000

**Table 4:** Model fit indices.

Fitting Index	CMIN/DF	RMR	RMSEA	GFI	AGFI	NFI	RFI	IFI	TLI	CFI
Criterion	<3	<0.05	<0.08	>0.9	>0.9	>0.9	>0.9	>0.9	>0.9	>0.9
Actual value	1.372	0.032	0.027	0.943	0.930	0.955	0.949	0.987	0.986	0.987

**Table 5:** Test of convergent validity.

Path			Estimate	S.E.	C.R.	*p*	Std. Estimate	CR	AVE
A3	←	AA	1				0.745	0.839	0.639
A2	←		0.899	0.059	15.327	***	0.685		
A1	←		1.268	0.064	19.730	***	0.945		
B4	←	BA	1				0.752	0.874	0.637
B3	←		1.054	0.060	17.577	***	0.770		
B2	←		0.963	0.058	16.688	***	0.735		
B1	←		1.225	0.058	21.138	***	0.921		
C4	←	CA	1				0.724	0.869	0.626
C3	←		1.120	0.069	16.331	***	0.756		
C2	←		1.099	0.066	16.601	***	0.768		
C1	←		1.306	0.068	19.207	***	0.904		
E5	←	EA	1				0.779	0.896	0.635
E4	←		0.933	0.054	17.277	***	0.728		
E3	←		1.035	0.054	19.128	***	0.791		
E2	←		0.955	0.054	17.778	***	0.745		
E1	←		1.209	0.052	23.114	***	0.926		
CT1	←	CT	1				0.922	0.894	0.629
CT2	←		0.798	0.040	20.035	***	0.721		
CT3	←		0.863	0.039	22.252	***	0.767		
CT4	←		0.855	0.038	22.416	***	0.770		
CT5	←		0.832	0.037	22.417	***	0.770		
PIN5	←	PIN	1				0.721	0.875	0.585
PIN4	←		1.041	0.064	16.155	***	0.749		
PIN3	←		1.004	0.064	15.739	***	0.730		
PIN2	←		0.920	0.061	15.098	***	0.701		
PIN1	←		1.218	0.063	19.252	***	0.905		

Note: *** means *p* < 0.001. AA—appearance anthropomorphism; BA—behavioral anthropomorphism; CA—cognitive anthropomorphism; EA—emotional anthropomorphism; CT—cognitive trust; PIN—purchase intention.

**Table 6:** Test of discriminant validity.

	1	2	3	4	5	6
1. Emotional Anthropomorphism	0.797					
2. Cognitive Anthropomorphism	0.687	0.791				
3. Behavioral Anthropomorphism	0.687	0.693	0.798			
4. Appearance Anthropomorphism	0.593	0.616	0.714	0.799		
5. Cognitive Trust	0.683	0.648	0.694	0.580	0.793	
6. Purchase Intention	0.656	0.605	0.632	0.625	0.650	0.765

**Table 7:** Path coefficients analysis.

Hypothesis	Estimate	S.E.	C.R.	*p*	β	Result
AA → CT	0.075	0.067	1.131	0.258	0.062	H1a: Not supported
BA → CT	0.366	0.077	4.749	***	0.314	H1b: Supported
CA → CT	0.234	0.076	3.086	0.002 **	0.181	H1c: Supported
EA → CT	0.346	0.064	5.387	***	0.306	H1d: Supported
AA → PIN	0.232	0.056	4.132	***	0.241	H2a: Supported
BA → PIN	0.057	0.065	0.886	0.375	0.062	H2b: Not supported
CA → PIN	0.091	0.063	1.457	0.145	0.089	H2c: Not supported
EA → PIN	0.217	0.055	3.922	***	0.243	H2d: Supported
CT → PIN	0.193	0.047	4.075	***	0.243	H3: Supported

Note: **, and *** mean *p* < 0.01 and *p* < 0.001. AA—appearance anthropomorphism; BA—behavioral anthropomorphism; CA—cognitive anthropomorphism; EA—emotional anthropomorphism; CT—cognitive trust; PIN—purchase intention.

**Table 8:** Mediation test.

		Bootstrapping		BC 95% CI		
		Est.	Std. Error	Lower Bound	Upper Bound	*p*-Value
Indirect effect	AA	0.015	0.016	−0.013	0.054	0.264
	BA	0.076	0.028	0.031	0.149	0.000
	CA	0.044	0.020	0.014	0.094	0.004
	EA	0.074	0.024	0.032	0.132	0.001
Direct effect	AA	0.241	0.062	0.117	0.360	0.001
	BA	0.062	0.079	−0.088	0.219	0.416
	CA	0.089	0.070	−0.049	0.226	0.191
	EA	0.243	0.067	0.097	0.363	0.002
Total effect	AA	0.256	0.064	0.128	0.384	0.001
	BA	0.139	0.078	−0.017	0.292	0.073
	CA	0.133	0.069	−0.004	0.266	0.056
	EA	0.317	0.066	0.176	0.435	0.002

Note: AA—appearance anthropomorphism; BA—behavioral anthropomorphism; CA—cognitive anthropomorphism; EA—emotional anthropomorphism; mediator—cognitive trust; dependent variable—purchase intention.

## Data Availability

Data are contained within the article. The original contributions presented in this study are included in the article. Further inquiries can be directed to the corresponding author.
